# Inhibition of mitochondrial dynamics in *Dictyostelium discoideum* through the overexpression of FszA and FszB proteins

**DOI:** 10.3389/fcell.2026.1903771

**Published:** 2026-09-04

**Authors:** Ashley Ryan, Kari Naylor

**Affiliations:** Department of Biology, University of Central Arkansas, Conway, AR, United States

**Keywords:** *Dictyostelium discoideum*, fission, FszA, FszB, FtsZ proteins, fusion, mitochondrial dynamics

## Abstract

Mitochondria play a central role in cells through energy production, calcium regulation, and cell death regulation. Dysfunction of mitochondria can impair energy production causing cellular damage which could be detrimental to an organism. Mitochondrial dynamics such as fission, fusion, and motility determine the organelle’s structure and can indicate the overall health of the cell. *Dictyostelium discoideum*, a well-established model for mitochondrial dynamics, contains two GTPase proteins that are predicted to mediate mitochondrial dynamics, FszA and FszB. In this study, we overexpressed GFP tagged FszA and FszB proteins to gain insight into their role in the mitochondrial dynamics of *D. discoideum*. Through live imaging, we quantified mitochondrial fission and fusion events, localization of the proteins with respect to fission and fusion events, and mitochondrial velocity. Results show that the overexpression of FszA-GFP, FszB-GFP, and GFP-FszB significantly decreased mitochondrial fission and fusion, and overexpressed GFP-FszA significantly decreased mitochondrial fusion compared to the control AX4 strain. Images of GFP-FszA and FszA-GFP strains showed little co-localization with the mitochondria during fission and fusion events, but GFP-FszB and FszB-GFP were localized to the mitochondria during fission events. Overexpression of FszB-GFP and GFP-FszB also significantly decreased mitochondrial velocity. The results of this study give insight into the underlying mechanism behind mitochondrial dynamics and could advance future studies in *D. discoideum* neurodegeneration models.

## Introduction

Mitochondria are organelles best known for their role in energy production through oxidative phosphorylation; however, they also play roles in programmed cell death through the release of cytochrome c and mediate metabolism through calcium regulation (Gunter and Gunter, 1994; Tian et al., 2022). Because of their roles in metabolism and cellular health, defects in mitochondrial function can lead to deficiencies in energy production in an organism (Wen et al., 2025).

Mitochondria undergo fission, fusion, and motility processes, collectively known as mitochondrial dynamics. Mitochondrial fission occurs when one mitochondrion splits into two separate mitochondria, and fusion occurs when two separate mitochondria combine to form one single organelle. Most organisms balance mitochondrial fission and fusion rates; however, depending on the cell type and the energy demands, these rates may vary between cell types and organisms (Friedman and Nunnari, 2014; Wisniewski et al., 2024). Mitochondrial dynamics play many roles in the cell including maintaining mitochondrial morphology, mitochondria distribution in a cell, and quality control of the mitochondria.

First, mitochondrial dynamics help maintain functional mitochondrial morphology. Mitochondria in yeast and mammalian COS-7 cells are more elongated and interconnected; however, dysfunctional fusion processes can cause mitochondria to become fragmented, and dysfunctional fission processes can cause very elongated mitochondria (Osteryoung and Nunnari, 2003). The dysfunctional mitochondrial morphology can lead to dysfunctional adenosine triphosphate production (Chen et al., 2023).

Secondly, mitochondrial dynamics are important to a cell because changes in energy demands across a cell might require a change in mitochondrial distribution. Functional transport of mitochondria across a cell is vital to cells, especially neurons. Neurons produce most of their mitochondria in the soma; however, mitochondrial products could be needed on the opposite side of the neuron at the synaptic terminal (Course and Wang, 2016). Neuronal mitochondria must be transported across the neuron making mitochondrial velocity and motility critical to neuronal health (Course and Wang, 2016).

Lastly, mitochondrial dynamics are used as quality control to ensure mitochondria do not accumulate dysfunctional products. If a mitochondrion has an error and produces a dysfunctional product, the mitochondrion can push the damaged products to one side of the organelle. The mitochondrion can then undergo fission separating the dysfunctional daughter mitochondria from the healthy daughter mitochondria. The dysfunctional mitochondria will go through mitophagy, and the healthy daughter mitochondria can remain functional (Youle and van der Bliek, 2012). In skeletal muscle cells, mitochondrial fusion can rescue mitochondrial function through the exchange of mitochondrial DNA (mtDNA) when mtDNA mutations arise (Chen et al., 2010; Friedman and Nunnari, 2014).

Because mitochondria play such a large role in the production of energy, calcium regulation, and programmed cell death of a cell, dysfunctional mitochondrial dynamics can lead to dysfunctional mitochondria function. While there is not a definitive cause of many neurodegenerative diseases, Parkinson’s disease (PD), Alzheimer’s disease (AD), and Huntington’s disease (HD), all show dysfunctional mitochondrial dynamics and morphology (Chen et al., 2023; Shirendeb et al., 2011; Wang et al., 2009). PD patients show increased expression of mitochondrial fission proteins leading to mitochondrial fragmentation (Chen et al., 2023). AD patients show abnormal mitochondrial distribution in neurons and decreased expression of both fission and fusion proteins (Wang et al., 2009). HD patients show increased expression in fission proteins and decreased expression in fusion proteins with fragmented mitochondria (Shirendeb et al., 2011). To fully understand mitochondrial fission and fusion processes and how to treat dysfunctional mitochondrial dynamics, researchers must fully understand the role of all proteins involved in mitochondrial dynamics.

Mammalian mitochondrial fission and fusion processes are carried out by dynamin-related proteins (DRPs) which are GTPase proteins that facilitate membrane remodeling (Hinshaw, 2000). Mammalian mitochondrial fission occurs when the endoplasmic reticulum marks the site of constriction on the mitochondria where mtDNA is replicating. Drp1, the main facilitator and DRP of mammalian mitochondrial fission, is then recruited to the constriction site and attaches to the receptors on the membrane. Endoplasmic reticulum bound with myosin IIa and actin as well as actin nucleating proteins will then constrict around the mitochondria until Drp1 can form a helix around the site. Drp1 hydrolyzes GTP, causing conformational changes and constricting the site until mitochondrial fission occurs (Tilokani et al., 2018).

Mammalian mitochondrial fusion is also carried out by DRPs, specifically mitofusions 1 and 2 (Mfn1/2) and OPA1 (Yu et al., 2020). Mfns located on the outer mitochondrial membrane will tether together when mitochondria encounter each other, pulling the membranes together and fusing the outer membranes. OPA1 carries out a similar fusion process on the inner mitochondrial membrane until the two inner membranes are fused into one, and two mitochondria have fused into one mitochondrion (Tilokani et al., 2018; Yu et al., 2020).

While mammalian mitochondrial fission and fusion are regulated by DRPs, there are additional membrane remodeling mechanisms found in other organisms (Hinshaw, 2000). Bacterial cells carry out their cell division using proteins known as FtsZ (filamentous temperature-sensitive mutant Z) proteins. FtsZ proteins are also GTPases that work by creating a cytokinetic ring inside the plasma membrane encircling the cell (Lutkenhaus and Addinall, 1997). This ring, known as a Z-ring, will undergo conformational changes as FtsZ proteins hydrolyze GTP constricting the Z-ring leading to bacterial cell division (Lutkenhaus and Addinall, 1997). FtsZ proteins are also used in chloroplast division (Osteryoung and Nunnari, 2003). Unlike bacterial division and mammalian mitochondrial division, chloroplasts use both DRP and FtsZ mechanisms (Chen et al., 2018; Osteryoung and Nunnari, 2003). In the inner chloroplast membrane, a Z-ring made of FtsZ proteins will form, and on the cytosolic side of the chloroplast, DRP5B proteins will create a helix. Both FtsZ proteins and DRPs will hydrolyze GTP and constrict both membranes at once until chloroplast division occurs (Chen et al., 2018).

Chloroplasts evolved from bacterial origins according to the endosymbiosis theory; therefore, it is logical for FtsZ proteins and DRPs to play a role in chloroplast division (Erickson, 2000; Gray, 2012). The mechanisms carried out by FtsZ proteins in eubacterium and archaebacterium are very similar to the mechanism DRPs carry out in mammalian mitochondrial fission; however, FtsZ proteins are not found in any animal genome (Tilokani et al., 2018). Because of the evolutionary link between bacteria, chloroplasts, and mammals, scientists hypothesize that FtsZ proteins found in prokaryotes were replaced with DRPs for eukaryotic mitochondrial fission (Erickson, 2000). In fact, the only eukaryotic organism to contain FtsZ proteins but not chloroplasts is *Dictyostelium discoideum* (Vaughan et al., 2004).

*D. discoideum* is a eukaryotic social amoeba that alternates between unicellular and multicellular forms depending on food availability and is an established model organism in evolutionary studies, mitochondrial disease studies, and neurological disorder studies (Storey et al., 2022). Researchers have even found *D. discoideum* to contain more orthologues to human disease genes compared to yeast, a more commonly utilized model organism (Eichinger et al., 2005). More specifically, *D. discoideum* has been found to contain homologues to human genes involved in AD, PD, and HD (Chen et al., 2023; Wang et al., 2009; Shirendeb et al., 2011). Because *D. discoideum* is a less complex eukaryote containing FtsZ proteins and homologues to neurodegenerative diseases, we can study *D. discoideum* mitochondrial dynamics to shed light on human neurodegenerative diseases linked to dysfunctional mitochondrial dynamics.

There have been a few studies conducted on the mitochondrial dynamics of *D. discoideum,* but the role of FtsZ proteins is still not fully understood. Woods et al. (2016) found that microtubules are the main cytoskeletal element used in *D. discoideum* mitochondrial dynamics while actin plays a smaller role (Woods et al., 2016). Gilson et al. (2003) studied the *D. discoideum* homologues of FtsZ proteins: FszA and FszB (Gilson et al., 2003). FszA and FszB are differentially localized to mitochondria suggesting a difference in the roles of the two proteins (Gilson et al., 2003). There has been little research on the role of FszA; however, it is predicted to be involved in mitochondrial fission as it has a potential mitochondrial import sequence and knock outs result in elongated mitochondria (Vaughan et al., 2004; Gilson et al., 2003). Vogel et al. (2020) overexpressed FszB and found that mitochondrial fission, fusion, and mitochondrial velocity decreased in *D. discoideum* (Vogel et al., 2020). The study presented here is a continuation of the Vogel et al. (2020) study with the acquisition of a new imaging system and overexpression of GFP (green fluorescent protein) tagged FszA and FszB proteins on both N- and C-terminals (Vogel et al., 2020).

The goal of this study was to continue to explore the role FszA and FszB proteins have in the mitochondrial dynamic processes of *D. discoideum,* through overexpression of GFP tagged FszA and FszB strains. Using live images, we quantified mitochondrial fission events, fusion events, and mitochondrial velocity. We also determined the localization of FszA and FszB during fission and fusion events to understand their role in the mitochondrial fission and fusion mechanism of *D. discoideum.* This data will help to establish if FszA and FszB are directly regulating mitochondrial dynamics through a physical association with the mitochondria or if they indirectly function through regulation of signaling pathways. Because *D. discoideum* contains homologues to human neurodegenerative disease genes, understanding the mitochondrial dynamics of *D. discoideum* could give researchers insight into the mechanisms of mitochondrial diseases in humans (Eichinger et al., 2005). These experiments may help direct the development of new therapies and treatments for patients diagnosed with neurodegenerative diseases that have been linked to dysfunctional mitochondrial dynamics as future studies in *D. discoideum* neurodegeneration models are carried out.

## Materials and methods

### Overview

**FIGURE 1:**
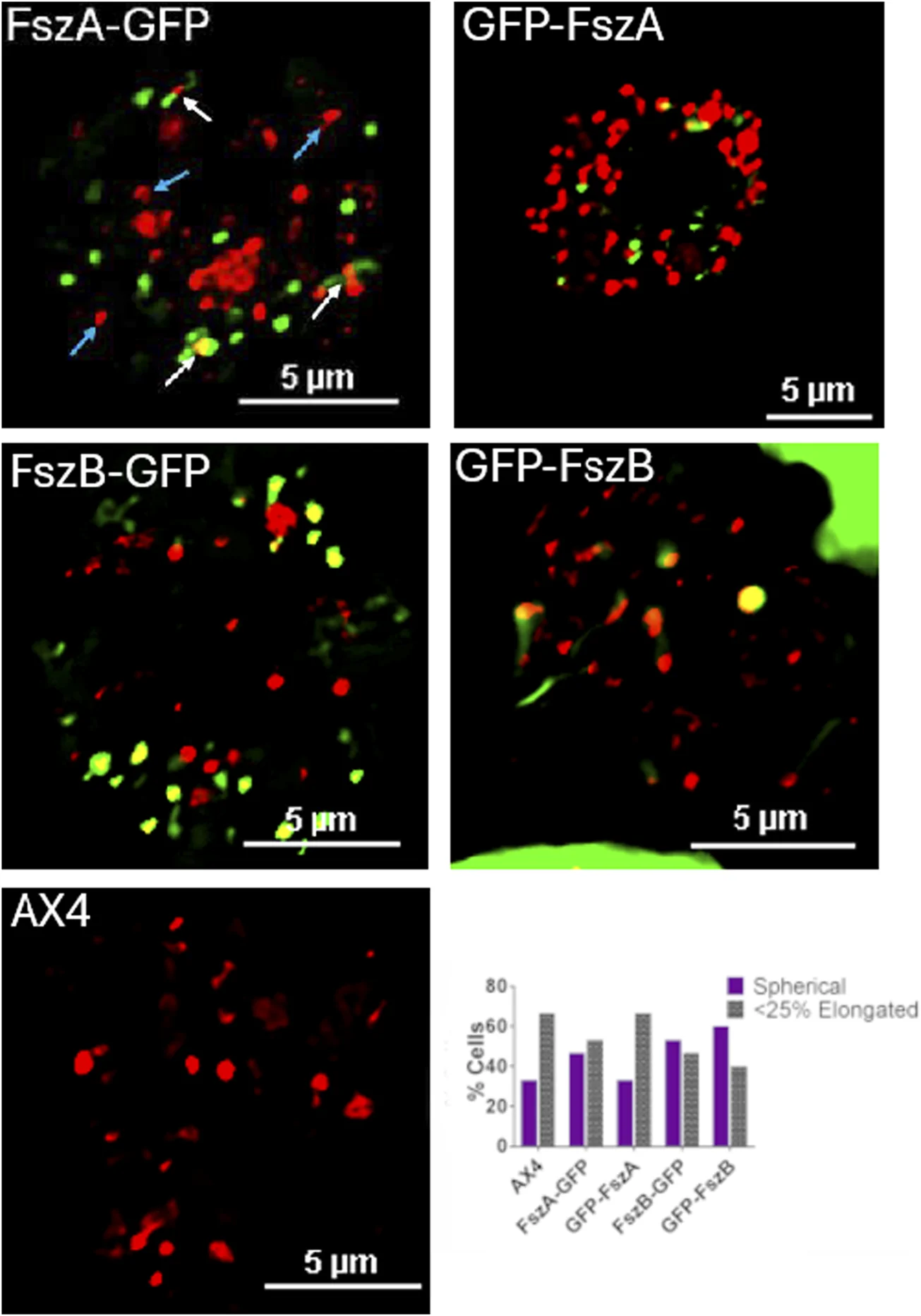
Expression and localization of GFP tagged proteins and mitochondrial morphology. Overexpression of FszA-GFP, GFP-FszA, GFP-FszB, and AX4 strains show less than 25% of mitochondria are elongated. Image of FszB-GFP overexpression shows all spherical mitochondria. FszA and FszB proteins are fluorescent green from the GFP tag and mitochondria are fluorescent red from Mitotracker. Arrows indicate how localization of the proteins on/touching the mitochondria were determined. The white arrows indicate FszA-GFP that is localized to the mitochondria, and the blue arrows indicate mitochondria without FszA-GFP. The graph shows percentage of cells in each morphology category. Statistically there is no difference across strains (p = 0.1420, n = 30 cells), but FszB strains do trend towards a more spherical morphology.

The goal of this study was to examine the role of FszA and FszB proteins in the mitochondrial dynamics of *D. discoideum* using the overexpression of GFP tagged FszA and FszB strains. *D. discoideum* axenic strain 4 (AX4) (wildtype) cells were transformed with different plasmids to create four overexpressed strains: FszA-GFP, GFP-FszA, FszB-GFP, and GFP-FszB. A GFP tag was used so the proteins could be visualized under fluorescent microscopy. The GFP tag was tested on both N- and C-terminals of FszA and FszB to ensure that the GFP tag itself was not disrupting the function of the proteins. After transformation, RT-PCR was performed to confirm overexpression of the proteins in the strains. Live cells were imaged using confocal microscopy to obtain 2-min videos of each strain (AX4, FszA-GFP, GFP-FszA, FszB-GFP, and GFP-FszB). These videos were then analyzed to compare the mitochondrial dynamics between AX4 and the overexpressed strains by analysis of mitochondrial morphology (Figure 1), mitochondrial fission and fusion rates, localization of FszA and FszB during fission and fusion events, and mitochondrial velocity.

#### Strain culturing and maintenance

The AX4 strain of *D. discoideum* was provided by Bill Loomis via Dicty Stock Center (www.dictybase.org/StockCenter/StockCenter.html) (Knecht et al., 1986). AX4 cells were cultured in HL5 media (10 mg/mL Oxford proteose peptone, 10 mg/mL dextrose anhydrous, 5 mg/mL yeast extract, 0.19 mg/mL NaH_2_PO_4_, 0.34 mg/mL KH_2_PO_4_) containing streptomycin (300 μg/mL) and ampicillin (150 μg/mL) at 22 °C with shaking at 119 rpm. Cells were diluted to 3 x 10^4^ cells/mL once they reached log-phase (1.0–2.0 x 10^6^ cells/mL), approximately every 3 days.

#### Plasmid creation

To create overexpressed FszA and FszB strains, plasmids were created. pDM323-FszA plasmid (FszA-GFP) was created by Azenta (Genewiz, NJ) by synthesizing the full length sequence of FszA, obtained from dictyBase (www.dictybase.org) with a 5′ BglII and Kozak sequence, AGATCTAAAAAA, and a 3′ flanking SpeI sequence, ACTAGT. The synthesized DNA was inserted into pDM323 (Veltman et al., 2009) via 5′ BglII and 3′ SpeI sites.

pDM317-FszA plasmid (GFP-FszA) was created by Azenta (Genewiz, NJ) by synthesizing the FszA gene sequence, obtained from dictyBase, without the start and stop codons and adding a 5′ BglII sequence, AGATCT, and 3′ SpeI sequence, ACTAGT. The synthesized DNA was digested and inserted into pDM317 (Veltman et al., 2009) via the BglII and SpeI sites.

pDM317-FszB (GFP-FszB) was created by Azenta (Genewiz, NJ) by synthesizing the FszB gene sequence, obtained from dictyBase, without the start and stop codons and adding a 5′ BamHI sequence, GGATCC, and a 3′ SpeI sequence, ACTAGT. The synthesized DNA was digested and inserted into pDM317 (Veltman et al., 2009) via the BamHI and SpeI sites.

pDXA3C-FszB-GFP (FszB-GFP) was obtained from Gilson et al., 2003; Gilson et al., 2003).

#### Transformation of strains

AX4 was transformed with pDM323-FszA-GFP, pDM317-GFP-FszA, pDM317-GFP-FszB (described above), or pDXA3C-FszB-GFP (Gilson et al., 2003). To transform AX4 cells, 1 x 10^7^ cells per reaction were centrifuged for 4 min at 500 x g. The cell pellet was washed 2 times with an equal volume of H-40 (40 mM HEPES, 1 mM MgCl_2_, pH 7), with final resuspension in 100 µL per reaction of H-40. 100 μL of the resuspended pellet and 2 μg of plasmid DNA was added to ice cold 2 mm cuvettes (Cat No. 1652086, Bio-Rad). Reactions were incubated on ice for 10 min.

Using the square wave protocol on the Bio-Rad Gene Pulser X-cell (Cat No. 1652666, 1652667, 1652668, and 1652669), cuvettes were pulsed with 2 pulses at 500 V for a pulse length of 0.05 millisecond with 2 s between each pulse. Cuvettes were incubated on ice for 15 min. Reactions were added to 10 mL of HL5 in a Petri dish, supplemented with 0.011 mM of folic acid (Sigma Cat No. F8758-5G) and 30 µL of 7-day conditioned HL5. Plates were incubated for 72 h at room temperature with no shaking.

To select for cells containing the plasmid, media was removed and fresh HL5 supplemented with folic acid, 7-day conditioned media, streptomycin (300 μg/mL), ampicillin (150 μg/mL), and G418 antibiotics (10 μg/mL) and incubated at 22 °C with no shaking. Media was changed every 3 days until cells in the control transformation (no plasmid DNA) died. New strains were diluted to 4 x 10^4^ cells/mL in HL5 supplemented with 300 μg/mL streptomycin, 150 μg/mL of ampicillin, and 10 μg/mL of G418 antibiotics. Once cells survived two dilutions growing in suspension, they were assayed for expression.

### Confirming overexpression of mRNA

To confirm the overexpression of mRNA, each strain underwent RT-PCR. RNA extraction was performed using the Zymo Research Quick-RNA Miniprep Kit (Cat No. R1054). cDNA was synthesized using the Thermo Scientific RevertAid First Strand cDNA Synthesis Kit (Cat No. K1621) by following manufacturer’s instructions using 500 ng of RNA and random hexamer primers. After cDNA was synthesized, the samples underwent PCR to amplify the DNA content, using primers in Table 1.

**TABLE 1:** Primers, sequences, and the expected cDNA and genomic band sizes. All four strains (FszA-GFP, GFP-FszA, FszB-GFP, and GFP-FszB) used control primers 1F and 2R which bind to the gene *RNLA,* a mitochondrial rRNA gene. 3F and 6R detect FszA, and 13F, 8R, 10R, and 12R detect FszB.

​	​	Expected sizes	
Primers	Sequence	cDNA	Genomic
1F	GGG​TAG​TTT​GAC​TGG​GGC​GG	350 bp	880 bp
2R	CAC​TTT​AAT​GGG​TGA​ACA​CC		
3F	TCA​GTT​CAT​GAT​TAG​ATA​TCA​AAT	829 bp	910 bp
6R	TGC​GAT​AGC​ATC​ACG​ACC​TTT		
8R	CAC​CAG​ATG​CTT​CAC​CAA​ATC​C	846 bp	1038 bp
10R	TCC​TCA​CCA​GAT​GCT​TCA​CCA		
12R	GCT​CTA​TCC​TCA​CCA​GAT​GCT​T		
13F	GAA​TTA​ACA​AAT​TGT​GGT​ATT​AG		

#### Live imaging of strains

Log-phase cells were pelleted and resuspended in an equal volume of Lo-Flo (Formedium Cat No. LF0501). 0.1 µM Mitotracker CMXRos (Invitrogen, Thermo Fisher Cat No. M7512) was added, and cells were incubated at 22 °C shaking at 119 rpm for 4 h, then washed twice with Lo-Flo. Using Nunc lab-TekII 4 well chambered cover glass (Cat No. 155382) cells were imaged with Nikon A1R confocal on a Ti2-E inverted microscope (Nikon, Melville, NY, USA), with a 4.6 AU pinhole, in 2-D every 0.1296 s timepoints (resonant scanner) for 2 min with 4x averaging. For each analysis, cells were imaged from 3–5 independent cultures.

#### Quantifying fission and fusion events in *Dictyostelium discoideum* strains

To quantify the number of mitochondrial fission and fusion events occurring in the strains, images were denoised, deconvoluted (Richardson-Lucy deconvolution with 20 iterations), and analyzed using NIS Elements AR 5.21.03 software (Nikon, Melville, NY, USA). Fluorescent intensity was manually configured for ease of analysis. A fission event was counted when one individual mitochondrion split into two mitochondria (Supplementary Movie 1). Fusion events were counted when two mitochondrion merged into one individual mitochondrion (Supplementary Movie 1). For both fission and fusion to be quantified, the mitochondria must have stayed merged as one or split as two individual mitochondria for multiple timepoints. Fission and fusion events were quantified in each cell per minute. Data analysis was conducted using the Wilcoxon matched pairs signed-rank test to compare AX4 fission to AX4 fusion or Kruskal–Wallis with Dunn’s *post hoc* analysis to compare average fission and fusion rates of 30 cells of each overexpressed strain to wildtype AX4.

#### Determining mitochondrial morphology in *Dictyostelium discoideum* strains

To determine if overexpressing FszA and FszB affected mitochondrial morphology in *D. discoideum* strains, images were analyzed using NIS Elements AR 5.21.03 software. Fluorescent intensity was manually configured for ease of analysis. Each cell’s mitochondrial morphology was determined to be either all spherical shaped mitochondria or mostly spherical shaped mitochondria with <25% elongated mitochondria. 30 cells per strain were analyzed for morphology and chi-square statistical analysis was used (Figure 1).

#### Determining mitochondrial velocity in *Dictyostelium discoideum* strains

Using cell tracking by NIS Elements AR 5.21.03 software, mitochondrial velocity was calculated from the same 30 cells of each strain. To ensure accurate velocity data per cell, cell tracking software was applied after single cells were cropped from each image. To isolate single cells, the transmitted light channel was removed. Then, under region settings, “use average background” and “smooth paste” were unchecked and gray set to 0. Images were converted to RGB, and again under region settings, “use average background” and “smooth paste” were unchecked and RGB values were each set to 0. A polygonal region was then drawn around a singular cell after selecting “Accepted Region” in the region settings of the edit section, and all frames were chosen. At this point, the surrounding background turned black except the region drawn around the single cell, and the mitochondrial velocity of a single cell could be calculated through the cell tracking software, by thresholding for mitochondria throughout the movie. Kruskal–Wallis with Dunn’s *post hoc* analysis was used to compare the average mitochondrial velocity per cell across 30 cells for each of the overexpressed strains to AX4, wildtype.

#### Determining localization of FszA and FszB with respect to fission and fusion events

Using just the overexpressed strains, fission and fusion events were identified in the first 30 s of the movies and determined if overexpressed GFP-FszA, FszA-GFP, GFP-FszB, or FszB-GFP were present, or enriched, on the mitochondria at the fission or fusion event. GFP structures appeared as green puncta while Mitotracker CMXRos labelled the mitochondria red. The presence of GFP structures localized on the mitochondria was indicated by a green GFP signal touching the red Mitotracker signal (Figure 1, Supplementary Movie 2). If a mitochondrion underwent fission or fusion and a green GFP signal was touching the red Mitotracker signal, this indicated that the protein was localized to the mitochondria during the event. If a mitochondrion underwent fission or fusion and a green GFP signal was not touching the red Mitotracker signal, at the event, the tagged protein was not localized to the mitochondria (Figure 1, Supplementary Movie 2). Fluorescent intensity was manually configured for ease of analysis. GFP structures were identified as present or absent from mitochondria during the fission and fusion event in 30 cells and analyzed via Wilcoxon matched pairs signed-rank test to determine if presence (or enrichment) on the mitochondria was due to random chance. While analyzing the GFP-FszB strain, GFP puncta were not always visible in the cell and instead sometimes showed as a GFP haze throughout the entire cell. Of the first 30 cells imaged of GFP-FszB (the same cells used for mitochondrial fission, fusion, and velocity analysis), 50% showed GFP puncta and 50% showed a GFP haze that was hard to interpret. To prevent misrepresentation of localization of GFP-FszB, the first 30 cells with visible GFP puncta were analyzed for localization instead of the first 30 cells that were imaged.

## Results

### Mitochondrial fission and fusion comparison in control AX4 *Dictyostelium discoideum* strain

**FIGURE 2:**
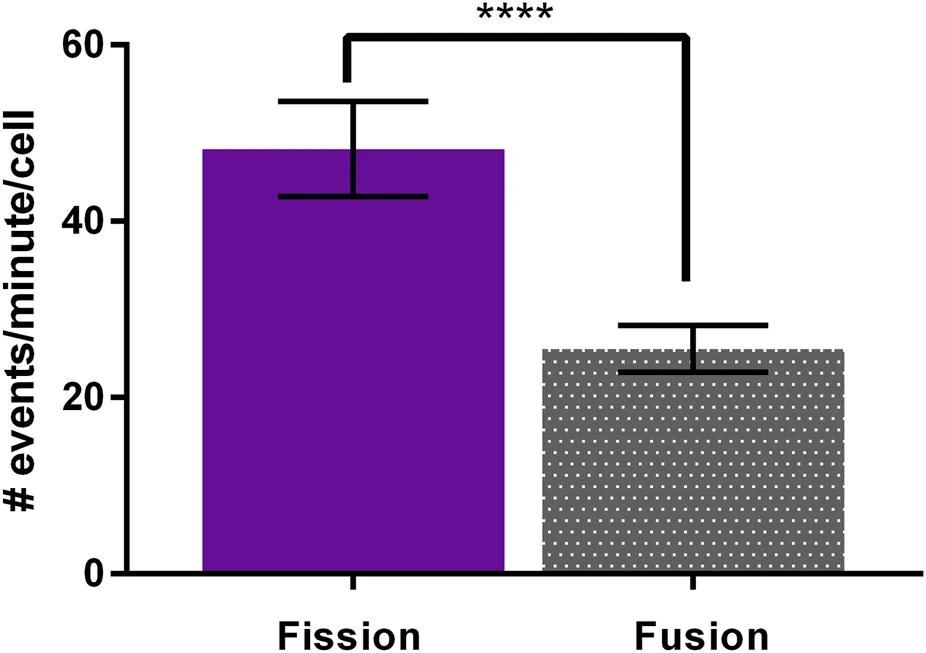
Quantified mitochondrial fission and fusion events in AX4 *Dictyostelium discoideum*. Two-minute time-lapse videos with 0.1296 s timepoints were manually analyzed using NIS Elements AR 5.21.03 software and Wilcoxon matched-pairs signed rank test to compare fission and fusion rates in AX4 strain (****p ≤ 0.0001; n = 30 cells). Fission rates were significantly increased compared to fusion rates in the AX4 strain. Columns indicate average and error bars are SEM.

Past research showed a balance between mitochondrial fission and fusion in AX4 *D. discoideum,* yet the organelle’s morphology suggests there should be more fission than fusion in this system (Schimmel et al., 2012). With the acquisition of a new imaging system with image deconvolution and higher temporal resolution (0.1296 s timepoints on new imaging system vs. 0.67738 s timepoints on old imaging system), quantification of fission and fusion shows a new statistically significant trend in untreated controls. Here we show an increased rate of mitochondrial fission (48.2 events/min) compared to fusion (25.5 events/min) in AX4 *D. discoideum* (Figure 2).

### Mitochondrial fission and fusion in *Dictyostelium discoideum* strains

**FIGURE 3:**
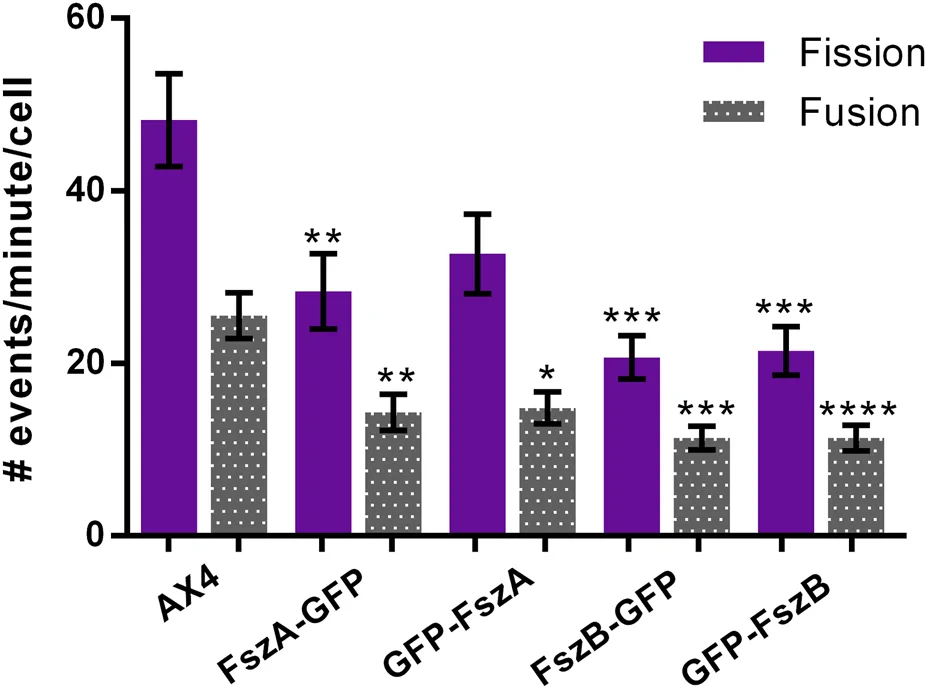
Quantified mitochondrial fission and fusion events in *Dictyostelium discoideum* strains. Two-minute time-lapse videos were manually analyzed using NIS Elements AR 5.21.03 software. Kruskal–Wallis with Dunn’s *post hoc* statistical analysis was used to compare the mitochondrial fission and fusion rates of the overexpressed *Dictyostelium discoideum* strains to the control AX4 strain (*p ≤ 0.05, **p ≤ 0.01, ***p ≤ 0.001, and ****p ≤ 0.0001; n = 30 cells). FszA-GFP, FszB-GFP, and GFP-FszB significantly decreased mitochondrial fission and fusion compared to AX4. GFP-FszA significantly decreased mitochondrial fusion compared to AX4. Columns indicate average and error bars are SEM.

To determine the role of FszA and FszB in mitochondrial dynamics, mitochondrial fission and fusion and morphology were quantified in overexpressed GFP tagged FszA and FszB strains and compared to the control AX4 strain (Figures 1, 3). Mitochondrial morphology was not significantly different between overexpressed strains and AX4 (Figure 1). On the other hand, fission and fusion events were significantly reduced in FszA-GFP, FszB-GFP, and GFP-FszB strains compared to the control AX4 strains. Only fusion was significantly reduced in the GFP-FszA strain compared to the control AX4 strain (Figure 3).

### Mitochondrial velocity in *Dictyostelium discoideum* strains

**FIGURE 4:**
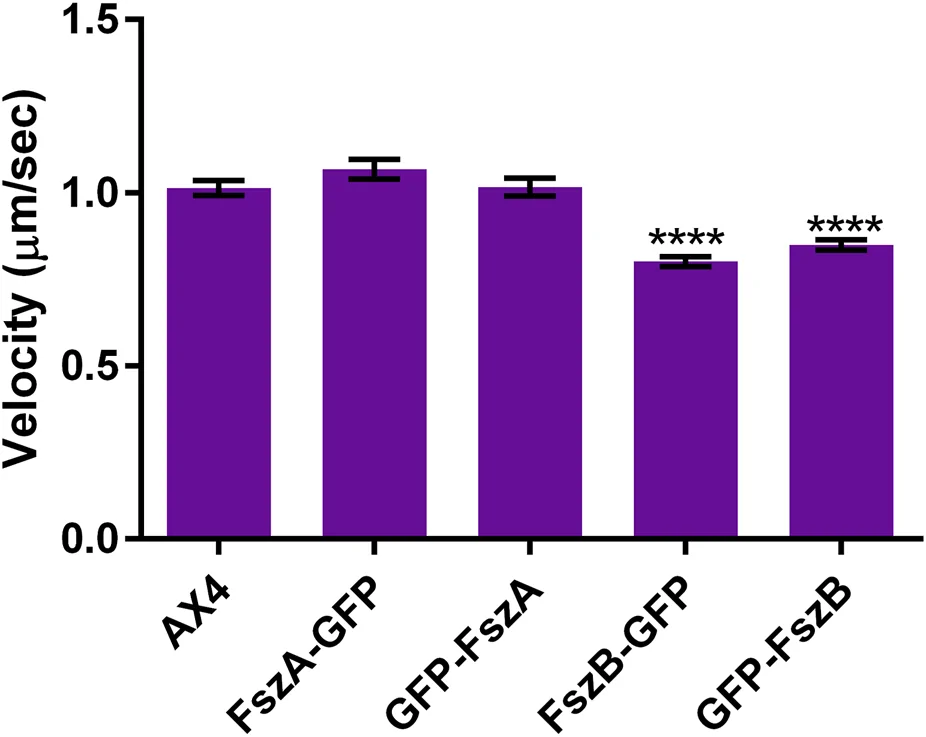
Mitochondrial velocity in *Dictyostelium discoideum* strains. Using cell tracking software by NIS Elements, mitochondrial velocity per cell was calculated from 2-min time-lapse videos of *Dictyostelium discoideum* strains. Kruskal–Wallis with Dunn’s *post hoc* analysis was used to compare average mitochondrial rates per cell between overexpressed strains and the control AX4 strain (****p ≤ 0.0001; n = 30 cells). There were no significant differences in mitochondrial velocity between AX4 and the overexpressed FszA strains (FszA-GFP and GFP-FszA). Overexpressed FszB strains (FszB-GFP and GFP-FszB) had significantly decreased average mitochondrial velocity per cell compared to the AX4 strain. Columns indicate average and error bars are SEM.

To determine if overexpressed FszA or FszB affects the motility of the mitochondria, mitochondrial velocity of the overexpressed GFP tagged FszA and FszB strains was tracked using NIS Elements cell tracking GA3 module and compared to mitochondrial velocity in the control AX4 strain (Figure 4). Mitochondrial velocity was significantly reduced in overexpressed FszB strains (FszB-GFP and GFP-FszB) compared to the AX4 strain. There was no difference in average mitochondrial velocity between overexpressed FszA strains (FszA-GFP and GFP-FszA) and the AX4 strain.

### Localization of FszA and FszB with respect to fission and fusion events in *Dictyostelium discoideum* strains

**FIGURE 5:**
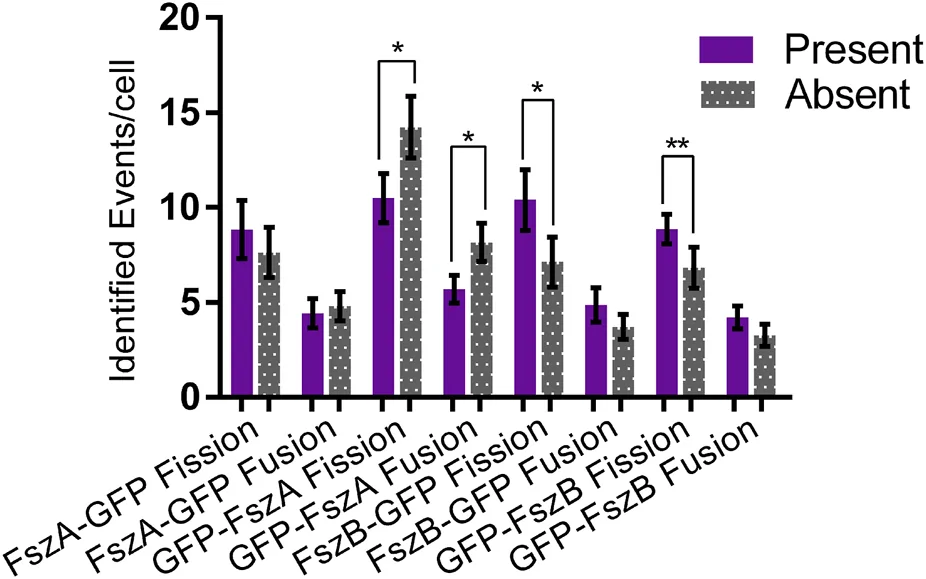
Localization of FszA and FszB with respect to fission and fusion events in *Dictyostelium discoideum* strains. GFP structures were determined to be “present” or “absent” at each fission and fusion event in 30 s time-lapse videos. The protein is considered present if the green GFP puncta touches the red mitochondria signal during the fission or fusion event. Results were analyzed via Wilcoxon matched pairs signed-rank test (*p ≤ 0.05, **p ≤ 0.01; n = 30 cells). GFP-FszA showed significant absence from mitochondria during fission and fusion events. FszA-GFP did not significantly show presence or absence of GFP structures on the mitochondria during fission or fusion events. FszB-GFP and GFP-FszB showed significant presence of FszB located on mitochondria during fission events. Columns indicate average and error bars are SEM.

To better understand FszA and FszB’s role in fission and fusion events, GFP structures were determined to be “present/enriched” or “absent” from the mitochondria during each fission and fusion event in time-lapse videos in the overexpressed strains (Figure 5, Supplementary Movie 2). GFP-FszA showed significant absence from mitochondria during fission and fusion events. FszA-GFP did not significantly show presence or absence of GFP structures on the mitochondria during fission or fusion events. FszB-GFP and GFP-FszB showed significant presence of FszB located on mitochondria during fission events.

## Discussion

### Mitochondrial fission and fusion in *Dictyostelium discoideum* strains

While analyzing mitochondrial fission and fusion results, we noticed a trend that was different from previous studies. Schimmel et al. (2012) found that mitochondrial fission and fusion rates were statistically balanced in wildtype AX4 *D. discoideum* with a rate of 1.00 ± 0.12 (mean ± SE) fission events per cell/minute and a rate of 0.95 ± 0.17 (mean ± SE) fusion events per cell/minute*;* however, our updated results using identical conditions show an imbalance in fission and fusion rates in our AX4 strain (Schimmel et al., 2012). Our results show a significant increase in mitochondrial fission compared to fusion. The difference in results could be due to the advancement in our imaging system with image deconvolution and higher temporal resolution resulting in clearer mitochondria structures and faster imaging compared to previous work (Schimmel et al., 2012). The structure of mitochondria in yeast and mammalian cells are elongated and highly connected with balanced mitochondrial fission and fusion rates; however, *D. discoideum* mitochondria are more spherical than elongated (Gilson et al., 2003; Osteryoung and Nunnari, 2003; Schimmel et al., 2012). An imbalance of fission and fusion rates typically changes the morphology of mitochondria. The significant increase in fission rates over fusion rates could lead to the more spherical mitochondria observed in *D. discoideum* AX4 strains.

### The role of FszA

Our results show that the overexpression of FszA-GFP significantly decreased mitochondrial fission and fusion rates compared to the AX4 control strain, while GFP-FszA significantly decreased fusion and decreased fission, but not significantly. Neither FszA overexpression strain significantly altered the velocity of mitochondria. Finally, the localization results of the overexpressed FszA strains during fission and fusion events showed varying results. FszA-GFP was not statistically present or absent on the mitochondria during either fission or fusion events. GFP-FszA was statistically absent from the mitochondria during fission and fusion events.

These results suggest that FszA could be an inhibitor of mitochondrial fission and/or fusion. This interpretation complements work done by Gilson et al. (2003) which found that overexpression of FszA-GFP resulted in more tubular mitochondrial morphology suggesting that the overexpression of FszA-GFP inhibited mitochondrial fission (Gilson et al., 2003). However, the results for FszA were not statistically identical across both the N-terminal and C-terminal tag, therefore we must consider the possibility that the GFP tag interferes with FszA function, possibly blocking the detection of the mitochondrial import sequence. From this work, FszA overexpression does not interfere with mitochondrial velocity and localization data suggests that FszA is not localized to the mitochondria during the fission and fusion events that do take place though results are not identical across both the N-terminal and C-terminal tag.

**FIGURE 6:**
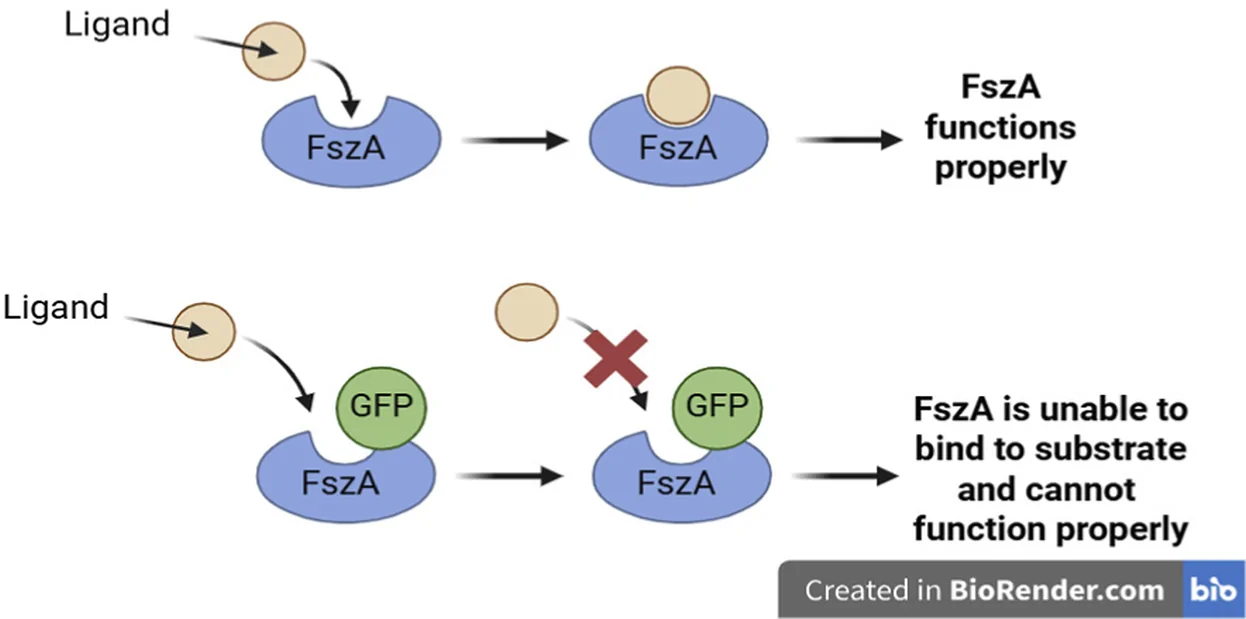
Model of GFP tag potentially interfering with FszA function. The large GFP tag on FszA might be blocking the binding site on FszA preventing FszA from functioning or localizing to the mitochondria properly. Schematic created in https://BioRender.com.

These results lead to two interpretations. First, this could suggest that FszA is part of a signaling pathway that could be inhibiting mitochondrial fission and fusion. This means that overexpression of the inhibitory signal decreases the rates of fission and fusion, and this protein does not localize to the mitochondria because it is regulating these processes from the cytosol rather than functioning as a mechanochemical enzyme. The second interpretation is that the GFP tag is interfering with FszA function because Gilson et al. (2003) found that knockouts of FszA and overexpression of FszA-GFP both created more elongated mitochondria morphology (Gilson et al., 2003). The inconsistent results for the N- and C-terminal tags from the fission and fusion analysis and the localization analysis, support this interpretation. Further support of this interpretation is that FLAG-FszA is enriched in mitochondrial fractions from AX4 *D. discoideum* mitochondrial isolations suggesting FszA does localize to mitochondria typically, but the GFP tag is interfering with this localization (Asamoah, 2025). The GFP tag is large (about 27 kD) and could be physically blocking a binding site on FszA (predicted to be about 53 kD) preventing the protein from functioning or localizing correctly (Figure 6) (Prozzillo et al., 2020; Gilson et al., 2003). Overall, our results suggest FszA might play a role in mitochondrial dynamics though future studies need to be conducted to ensure the GFP tag is not interfering with the function of the protein.

### The role of FszB

Our analysis of overexpressed GFP tagged FszB shows that both constructs significantly inhibited mitochondrial fission and fusion compared to the AX4 control strain. Both FszB-GFP and GFP-FszB constructs significantly decreased mitochondrial velocity compared to the AX4 control strain and are localized to the mitochondria during fission events.

These results suggest that FszB could play a mechanical role at the site of fission and overexpression of FszB might physically inhibit mitochondrial dynamics. The results presented here are supported by previous work from our lab studying only the C-terminal GFP tag of FszB via an older imaging system. Vogel et al. (2020) found that overexpression of FszB-GFP inhibits mitochondrial fission, fusion, velocity and localizes to the mitochondria during fission events; but also, during fusion events which does not match results presented here (Vogel et al., 2020). The difference in the localization results could be due to the advancement in imaging that was used in these experiments, but FszB trends towards its presence at fusion events even though in this study it is not statistically significant. In terms of velocity, overexpression of FszB inhibits fission/fusion which may alter the capacity for mitochondrial movements, or *vice versa*. Woods et al. (2016) found that microtubules are the main element responsible for the movement of mitochondria (Woods et al., 2016). They also found actin to play a smaller role in mitochondrial movement as disruption of actin led to a decrease in the number of mitochondria moving, but not in the velocity of the moving mitochondria (Woods et al., 2016). This supports our conclusion that overexpression of FszB inhibits not only fission and fusion but also velocity. It is possible that FszB inhibits mitochondria from loading onto microtubules or could be directly inhibiting membrane remodeling when overexpressed and this indirectly inhibits mitochondrial motility. Additional work needs to be done to determine FszB’s exact method of inhibition, including following up on the possibility of overexpression altering membrane potential which may confound the results presented here.

### Future studies

To better understand the role FszA plays in the mitochondrial dynamics of *D. discoideum* additional tags should be studied with overexpressed FszA strains. The large GFP tag might be interfering with the role FszA is playing in mitochondrial dynamics due to the inconsistent results from N- and C-terminus tags in fission, fusion, and localization analyses (Figure 6). An overexpressed FszA strain with a smaller tag such as the FLAG tag (8 amino acids) on the N- and C-terminus may not interfere with the protein’s role and could give more insight into the function of the FszA protein (Prozzillo et al., 2020). To better understand the localization of FszA during mitochondrial dynamics, immunofluorescence of FLAG tagged FszA strains should be analyzed. Developing a knockdown of FszA is also critical to gain insight into FszA function; however, the development of a stable knockdown strain has proven to be challenging.

After this study, the role of FszB in mitochondrial dynamics seems to be clearer; however, following up with future studies would solidify our understanding of FszB. Based on the results in this experiment, the GFP tag in the overexpressed FszB strains did not interfere with the function of FszB, but analysis of a FLAG-FszB strain should be undertaken to confirm the conclusions from this work are accurate. Developing a knockdown of FszB and repeating these analyses would further help scientists understand the role of FszB in mitochondrial dynamics. We would expect that knocking down FszB could increase fission and fusion events and increase the mitochondrial velocity in *D. discoideum.*

An additional caveat that was not considered by this study is the possibility that the overexpression of FszA or FszB alters the potential of the mitochondrial membrane. Mitotracker CMXRos is dependent upon membrane potential to label mitochondria, therefore it is possible that unlabeled mitochondria were not analyzed in these assays. An analysis of these strains for membrane potential should be carried out, to strengthen the conclusions drawn here.

## Conclusion

Our results provide insight into the molecular mechanism of mitochondrial dynamics in *D. discoideum.* This study suggests that FszA might play a role in mitochondrial dynamics, though future studies need to be conducted to ensure the GFP tag is not interfering with the function of the protein. Our results suggest that FszB could be physical inhibitor of fission and fusion which is supported by other studies (Vogel et al., 2020). Because FszB also inhibits mitochondrial velocity, we believe that FszB could physically inhibit mitochondria from loading onto the cytoskeleton to undergo normal mitochondrial movement, fission, and fusion. This would explain why the overexpression of FszB led to decreased velocity, fission events, fusion events, while FszB was localized to the mitochondria, though additional studies need to be conducted to support this conclusion.

Though our study was conducted in *D. discoideum,* understanding the mechanism of mitochondrial dynamics in non-mammalian eukaryotes could shed light on the mechanism in humans. Many neurodegenerative diseases such as AD, PD, and HD exhibit dysfunctional mitochondrial dynamics, and there are limited treatments and therapies for these patients; however, some treatments do target DRPs due to the dysfunctional mitochondrial dynamics (Chen et al., 2023; Meng et al., 2025). Our study improves our understanding of mitochondrial dynamics and continued studies, such as testing treatments in *D. discoideum* disease models, could provide additional understanding of neurodegenerative diseases and their treatments.

## Data Availability

The raw data supporting the conclusions of this article will be made available by the authors, without undue reservation.
