# Supplementary material for: Inhibition of mitochondrial dynamics in Dictyostelium discoideum through the overexpression of FszA and FszB proteins
Source: Front Cell Dev Biol. 2026 Sep 4;14:1903771. doi: 10.3389/fcell.2026.1903771 (PMC13585573; doi:10.3389/fcell.2026.1903771)
Supplement: Supplementary file 1 [file Supplementaryfile1.docx]

Supplemental Movie 1

Movie at actual speed of AX4 cell labelled with MitotrackerCMXRos. Gray arrows indicate fission events. Hand drawn white arrows indicate fusion events.

Supplemental Movie 2

Movie at actual speed of an AX4 cell overexpressing FszB-GFP and labelled with MitotrackerCMXRos. White asterisk indicates fusion with FszB-GFP present. White arrow with black outline indicates fusion without FszB-GFP. Red hand drawn arrow indicates fission event with FszB-GFP. White arrow indicates fission without FszB-GFP.
